# Association of Kidney Stone Disease (KSD) with Primary Gastrointestinal Surgery: a Systematic Review over Last 2 Decades

**DOI:** 10.1007/s11934-021-01046-w

**Published:** 2021-05-24

**Authors:** Y. Premakumar, N. Gadiyar, B. M. Zeeshan Hameed, D. Veneziano, B. K. Somani

**Affiliations:** 1 Guys and St Thomas NHS Trust, London, UK; 2 Croydon University Hospital NHS Trust, London, UK; 3 Department of Urology, Kasturba Medical College Manipal, Manipal Academy of Higher Education, Manipal, Karnataka India; 4 Department of Urology and Kidney transplant, Grande Ospedale Metropolitano di Reggio, Reggio di Calabria, Italy; 5 University Hospital Southampton NHS Trust, Southampton, UK

**Keywords:** Kidney stones, Urolithiasis, GI surgery, Obesity, Bariatric surgery, Bowel surgery, Inflammatory bowel disease

## Abstract

**Purpose of review:**

We aim to provide an up-to-date literature review to further characterise the association of kidney stone disease (KSD) with gastrointestinal (GI) surgery. As KSD is associated with significant morbidity, it is important to quantify and qualify this association to provide better care and management for the patient subgroup.

**Objective:**

To perform a systematic review of the existing literature to evaluate the association of KSD following GI surgery.

**Methods:**

A literature search was performed of the following databases: MEDLINE, EMBASE, Scopus, Google Scholar, Key Urology, Uptodate and Cochrane Trials from January 2000 to June 2020.

**Recent Findings:**

A total of 106 articles were identified, and after screening for titles, abstracts and full articles, 12 full papers were included. This involved a total of 9299 patients who underwent primary GI surgery. Over a mean follow-up period of 5.4 years (range: 1–14.4 years), 819 (8.8%) developed KSD, varying from 1.2 to 83% across studies. The mean time to stone formation was approximately 3 years (range: 0.5–9 years). In the 4 studies that reported on the management of KSD (*n* = 427), 38.6% went on to have urological intervention.

**Summary:**

There is a high incidence of KSD following primary GI surgery, and after a mean follow-up of 3 years, around 9% of patients developed KSD. While the GI surgery was done for obesity, inflammatory bowel disease or cancer, the risk of KSD should be kept in mind during follow-up, and prompt urology involvement with metabolic assessment, medical and or surgical management offered as applicable.

## Introduction

Surgery is an important aspect of managing an increasing number of gastrointestinal (GI) diseases. Specifically, it is a common management option for inflammatory bowel disease (IBD) and obesity. It is said that up to 70% of patients with Crohn’s disease and 35% of patients with ulcerative colitis (UC), the two entities comprising IBD, will require surgery at some point during their disease [1]. Similarly, in bariatric surgery for obesity, the surgical options consist of restrictive methods (e.g. banding), creating a state of malabsorption by using bypasses or a mixed approach (e.g. Roux-en-Y gastric bypass). Obesity is becoming an important and overwhelming health problem with an estimated 28% of adults in the UK, and 42.4% of adults in the USA considered obese [2, 3]. As the rate of obesity increases, so will the number of bariatric surgery procedures [2, 4].

It is well-recognised that there is an association between kidney stone disease (KSD) and these malabsorptive states; however, there is little information regarding the incidence of KSD following these surgeries [5••, 6••]. Furthermore, it is not known whether these malabsorptive states follow all GI surgeries and what the nature of this association is.

The aim of this systematic review was to evaluate the epidemiological evidence of KSD following GI surgery in adult patients, specifically looking at incidence and time to stone formation, types of GI surgery, and management of stones. Based on the review, we have also summarised the key findings for clinical practice.

## Methods

### Search Strategy

**Fig. 1:**
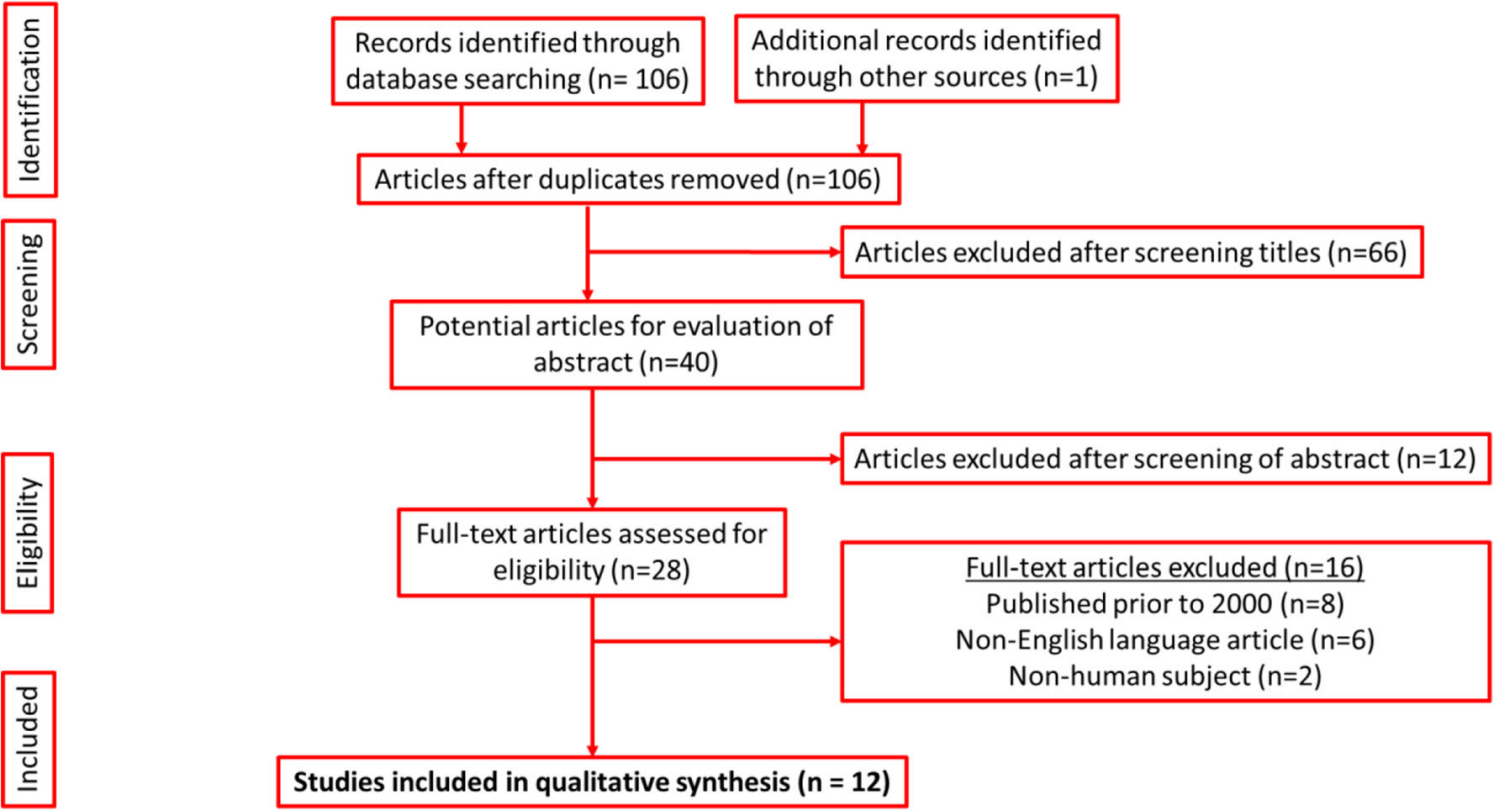
PRISMA diagram outlining search results. Diagram reproduced from: http://prismastatement.org/PRISMAStatement/FlowDiagram

A protocol of the review was drafted and approved by PROSPERO (ID: 180713) prior to commencing the study. This systematic review has been performed according to the Cochrane style and in accordance with the Preferred Reporting Items for Systematic Reviews and Meta-Analysis (PRISMA) checklist. A search of MEDLINE, EMBASE, Scopus, Google Scholar, Key Urology, Uptodate and Cochrane Trials was performed from January 2000 to June 2020 (Fig. 1). Search terms included the following words and combinations of words: “renal stones”, “kidney stones”, “urolithiasis”, “nephrolithiasis”, “colectomy”, “laparotomy”, “bariatric surgery”, “obesity surgery”, “bowel surgery”, “inflammatory bowel disease”, “bowel cancer”, “gastric surgery” and “gastric bypass”. Reference lists from papers identified were then scanned for further relevant papers.

### Evidence Acquisition: Criteria for Considering Studies

#### Inclusion Criteria

1. Studies reporting on primary GI surgical procedures (defined as surgical procedures involving GI tract for GI pathology)
2. Studies reporting on a minimum of 20 patients
3. English language studies
4. Adult patients (≥ 18 years of age)

#### Exclusion Criteria

1. Laboratory, animal data or review articles
2. Studies published before 2000

### Data Extraction and Analysis

Studies meeting the inclusion criteria had data extracted by two independent reviewers (YP,NG) using a data form designed for this review. Data extracted included number of patients, age, male:female ratio, diagnosis of bowel pathology, time from diagnosis of bowel pathology to stone formation, location of stone, size of stone, stone demographics, management and follow-up of stone and recurrence of stone (number, location, time until recurrence). Discrepancies in data collection were discussed and resolved by the senior author (BS).

Study quality was assessed using the validated GRADE framework as it allows for bias assessment in a variety of study types [7]. Studies with a high risk of bias were to be excluded; none was identified during this review.

## Results

Our search methodology produced a total of 106 articles, of which 12 met the inclusion criteria and were subsequently included in the final analysis (Fig. 1).

### Characteristics of Included Studies

There are 5 case series [8–12], 3 retrospective cohort studies [13, 14•, 15], 3 prospective cohort studies [16•, 17•, 18] and 1 case-control study [19•] (Tables 1 and 2). Majority of the studies took place in the USA (*n* = 9) with two studies authored in Brazil and one in Japan.

**Table 1:** Baseline characteristics for all studies included in the quantitative synthesis of KSD incidence

Lead author (year) Country	Study type	*N* = total (# of pts who developed KSD)		Mean age (range)		Male: female		Type of bowel procedure	Definition of control	Diagnosis of KSD ascertainment	Follow-up time (years)
		P	C	P	C	P	C				
Parks (2003) USA	Case-control	180 (150)	1688 (44)	-	44	140:40	-	Bowel surgery for bowel pathology (included IBD, trauma, cancer, bypass procedures for obesity or hypercholesterolemia). Defined as large bowel, small bowel, both or bypass	Patients who are stone formers with no bowel pathology	Review of medical records, radiographs and stone analyses	6.8
Durrani (2006) USA	Ret. cohort	972 (31)	-	-	-	-	-	RYGB	-	Review of medical records, imaging	7
Sinha (2007) USA	Case series	1436 (60)	-	48 (30-61)	-	-	-	RYGB	-	Not stated	2.
Matlaga (2009) USA	Ret. cohort	4639 (355)	4639 (215)	44.6	45.0	905:3734	905:3734	RYGB for obesity	Obese patients who did not have surgery	Coding of medical records	5
Semins (2009) USA	Ret. cohort	201 (3)	201 (12)	46.3	46.5	35:166	35:166	Gastric banding	Obese patients who did not have surgery	Coding of medical records	2.3
Penniston (2009) USA	Case series	39	-	51.2 (25-75)	-	11:28	-	RYGB (*n* = 27) and gastric banding (*n* = 12) for obesity	-	Clinical history	3
Costa-Matos (2009) Brazil	Case series	58	-	39.3 (19-63)	-	5:24	-	RYGB for obesity	-	Medical records, US imaging (6 monthly, 1 yearly)	14.4
Mukewar (2013) USA	Prosp. cohort	218 (64)	137	49.1 (12.4 SD)	46.5 (14.7)	5:3	73:64	UC patients with ileal pouch-anal anastomosis (IPAA)	UC patients with IPAA, no history of KSD and no KSD on post-colectomy imaging	Clinical history and CT,MRI or US imaging	10
Shimizu (2013) Japan	Prosp. cohort	226	-	65 (36-87)	-	19:10	-	Gastric cancer patients for distal gastrectomy with Billroth-I (*n* = 60), or with R-Y (*n* = 81) reconstruction; and total gastrectomy with R-Y reconstruction (*n* = 85)	-	CT imaging yearly with various timing based on cancer grade	3
Valezi (2013) Brazil	Case series	151 (11)	-	-	-	-	-	RYGB for obesity	-	Clinical history and US imaging 1 year post-operatively	1
Chen (2013) USA	Ret. case series	417 (5)	-	-	-	1:4	-	Gastric banding (*n* = 332) and sleeve gastrectomy (*n* = 85) for obesity	-	Coding, CT or US imaging	5
Lieske (2015) USA	Prosp. cohort	762	759	44.7 (11.2 SD)	44.7 (11.2 SD)	148:614	148:611	RYGB (*n* = 592), very very long limb RYGB (*n* = 55), biliopancreatic diversion/duodenal switch (*n* = 50), banding (*n* = 43) or sleeve gastrectomy (*n* = 13)	Obese pts (BMI > 35) who did not undergo surgery	Not stated	6

*P* underwent procedure, *C* control, *IBD* inflammatory bowel disease, *RYGB* Roux-en-Y gastric bypass, *UC* ulcerative colitis, *US* ultrasound, *CT* computed tomography, *KSD* kidney stone disease, *Ret.* retrospective, *Prosp.* prospective, *N/A* not applicable

**Table 2:** KSD incidence outcomes

Lead author (year)	*N* = total		Mean age (range)		Male:female		Type of bowel procedure	Follow-up time (years)	# pts developing KSD (%)		Time to stone formation (years)	Management of KSD
	P	C	P	C	P	C			P	C		
Parks (2003)	180	1688	-	44	140:40	-	Bowel surgery for bowel pathology (included IBD, trauma, cancer, bypass procedures for obesity or hypercholesterolaemia). Defined as large bowel, small bowel, both or bypass	6.8	150 (83%)	44 (2.6%)	9	Not stated
Durrani (2006)	972	-	-	-	-	-	RYGB	7	31 (3.1%)	-	2.8	Not stated
Sinha (2007)	1436	-	48 (30-61)	-	-	-	RYGB	2.	60 (4.1%)	-	2.9	Not stated
Matlaga (2009)	4639	4639	44.6	45.0	905:3734	905:3734	RYGB for obesity	5	355 (7.6%)	215 (4.6%)	1.5	153 underwent urological procedure (98 Ureteroscopy +/- lithotripsy, 81 lithotripsy, 6 PCNL)
Semins (2009)	201	201	46.3	46.5	35:166	35:166	Gastric banding	2.3	3 (1.5%)	12 (6.0%)	-	1 ureteroscopy, 0 PCNL, 0 lithotripsy
Penniston (2009)	39	-	51.2 (25-75)	-	11:28	-	RYGB (*n* = 27) and gastric banding (*n* = 12) for obesity	3	8 (20.5%)	-	-	Not stated
Costa-Matos (2009)	58	-	39.3 (19-63)	-	10:48	-	RYGB for obesity	14.4	1 (1.7%)	-	0.5	Not stated
Mukewar (2013)	218	137	49.1(12.4 SD)	46.5 (14.7 SD)	40:24	73:64	UC patients with ileal pouch-anal anastomosis	10	64 (29.3%)	-	-	11 had Urology review, 9 had urological procedures
Shimizu (2013)	226	-	65 (36-87)	-	19:10	-	Gastric cancer patients for distal gastrectomy -with Billroth-I (*n* = 60), or with R-Y (*n* = 81) reconstruction; and total gastrectomy with R-Y reconstruction (*n* = 85)	3	31 (13.7%)	-	1.4	Not stated
Valezi (2013)	151	-	-	-	-	-	RYGB for obesity	1	27 (17.9%)	-	-	Not stated
Chen (2013)	417	-	-	-	1:4	-	Gastric banding (*n* = 332) and sleeve gastrectomy (*n* = 85) for obesity	5	5 (1.2%)	-	2.85	2/5 had urology procedure (1 PCNL, 1 lithotripsy), 1 passed spontaneously
Lieske (2015)	762	759	44.7 (11.2 SD)	44.7 (11.2 SD)	148:614	148:611	RYGB (*n* = 592), very very long limb RYGB (*n* = 55), biliopancreatic diversion/duodenal switch (*n* = 50), banding (*n* = 43) or sleeve gastrectomy (*n* = 13)	6	84 (11%)	33 (4.3%)	-	Not stated

*P* underwent procedure, *C* control, *IBD* Inflammatory bowel disease, *RYGB* Roux-en-Y gastric bypass, *UC* ulcerative colitis, *KSD* kidney stone disease, *PCNL* percutaneous nephrolithotomy

A total of 9299 patients who underwent a primary GI surgery were included. The mean age of patients was 48.5 years (range: 44.6–65), with a male:female ratio of 1289:4118 (noting that three studies did not report sex characteristics). The mean follow-up time of patients was 5.4 years (range: 1–14.4 years). Due to the heterogeneity of studies, further analysis on the data was limited, and a narrative synthesis of available data took place.

### Types of GI Procedures

The majority of GI procedures was for obesity, with 8 papers including bypass surgeries and 4 including gastric banding procedures. Roux-en-Y gastric bypass (RYGB) was the most popular bypass procedure, followed by sleeve gastrectomy and biliopancreatic diversion/duodenal switch.

Shimizu was the only paper to cover types of gastrectomies performed for gastric cancer [17•]. They included distal gastrectomy with Billroth-I (DGBI) (*n* = 60) or Roux-en-Y (DGRY) (*n* = 81) reconstruction and total gastrectomy with Roux-en-Y (TGRY) reconstruction (*n* = 85). KSD was detected in 3 DGBI patients (5%), 7 DGRY patients (8.6%), and 21 TGRY patients (24.7%). They found significant differences in the frequency of KSD between DGBI and TGRY (*p* = 0.004) and between DGRY and TGRY (*p* = 0.011) patients, but not between DGBI and DGRY groups. In addition to detection of KSD, renal dysfunction was assessed with serum creatinine measurements and found in 5 TGRY patients, all of whom were found to have KSD. One of these patients with renal dysfunction went on to have a renal biopsy which found diffuse interstitial fibrosis and tubular degenerative changes with intraluminal crystals. Stone analysis revealed primarily calcium oxalate stones.

Only two papers investigated GI surgery for IBD. Parks looked at bowel surgery for a variety of indications which included obesity and IBD, cancer and trauma and looked specifically at small/large bowel resections or bypasses (*n* = 2228) [19•]. Mukewar looked at ileal pouch-anal anastomosis (IPAA) in ulcerative colitis (UC) patients (*n* = 64) [16•].

### Diagnosis of KSD

Ascertainment of KSD diagnosis ranged from clinical history to different imaging modalities. The most popular method was imaging (*n* = 5), of which two studies relied on ultrasound (US) [10, 11]; only one study relied on computed tomography (CT) only [17•]; and two studies relied on a combination of US or CT or magnetic resonance imaging [12, 16•]. Other studies mentioned the identification of patients via coding [14•, 15, 16•] or clinical history [9], but they did not state the imaging modality of diagnosis, which is presumed to be during follow-up of their primary GI pathology.

The percentage of patients who developed KSD post-procedure varied between studies from 1.2 to 83%. Of 9299 patients who underwent a primary GI procedure, a total of 819 patients developed KSD (8.8%). Mean time to stone formation was approximately 3 years (range 0.5–9 years).

Parks reported KSD following bowel surgery in 180 patients, where the primary pathology was IBD, cancer trauma or obesity-related procedure [19•]. They provided details of KSD post-procedure for the following bowel surgeries: large and small bowel resection (LB&SB), large bowel resection only (LB), small bowel resection only (SB) and bypass procedures. The number of patients developing KSD following these procedures and the average time taken until stone formation were reported as: 29 (16.1%) LB&SB patients took 13 years (+/-2 years) for stone formation, 58 (32.2%) LB patients took 6 years (+/-1 year) for stone formation, 36 (20%) SB patients took 11 years (+/-1 year) for stone formation and 27 (15%) bypass patients taking 6 years (+/-2 years) for stone formation.

### Studies Which Included Controls

There was great variability in what studies deemed “controls”. Parks included patients who were known stone formers without any bowel pathology [19•]. Three studies used matched obese patients who did not have surgery; however, it is not clear if these patients were known to be stone formers or not [14•, 15, 18]. Overall, the variability in defining controls made it difficult to assimilate control data. When comparing KSD in controls versus patients who underwent GI surgeries, there was no obvious relationship.

### Management of KSD

Management of KSD was documented for 4 studies in our review. In the series from Matlaga, of the patients who developed KSD, 153 (43.1%) patients underwent a urological procedure [14•]. Of the 64 patients who developed KSD in another study, 11 were reviewed by urology, and 9 (14%) proceeded to have unspecified urological procedures [16•]. Chen and Semins reported lower incidences of KSD than other papers that reported on management [12, 15]. Chen reported that 2 of the 5 patients who developed KSD post-procedure underwent a urological procedure: 1 lithotripsy and 1 percutaneous nephrolithotomy (PCNL) [12]. Semins reported that 1 of the 3 patients developed KSD post-procedure, and they subsequently underwent an ureteroscopy [15].

Overall from these 4 studies, 38.6% of patients who were diagnosed with KSD following their bowel surgery procedure (*n* = 427) went on to have a urological procedure for management of their KSD.

## Discussion

### Meaning of the Study

The aim of this systematic review was to provide an updated summary on the association between KSD following primary GI surgeries. We have summarised the most up-to-date information available and have expanded on previous work done by Gonzales [5••] and Gkentzis [6••]. We demonstrate a positive association of KSD following GI surgeries, with a mean incidence rate of 8.8% and a mean time of 3 years (range 0.5–9 years) to stone formation. The most frequent type of GI procedure reported on was bariatric surgeries: bypass and banding surgeries. The incidence for KSD following GI procedures for other indications (e.g. cancer and IBD) was reported infrequently; however, these few papers reported KSD incidence rates > 10% on average [16•, 17•, 19•].

### Bariatric Surgery and Kidney Stone Disease

KSD following RYGB was first described in 2005 by Nelson when it was noted there was a significant proportion of patients developing calcium oxalate stones [20]. The pathophysiology behind this is an increase in urinary oxalate excretion, a known risk factor for KSD, thought to be due to malabsorption of bile salts and fatty acids by dysfunctional or absent segments of GI tract. This is then exacerbated by GI surgery where segments are resected or rendered dysfunctional. This state of hyperoxaluria leads to urinary calcium oxalate supersaturation which then leads to crystal aggregation and stone formation [10, 21, 22]. It is also reported that the other factors which increase the risk of KSD in this population are low volume and hypocitraturia. Parks contended that chronic acidosis in the urine predisposes this population to hypocitraturia, and thus stones, as urinary citrate, are a known stone inhibitor [23].

Of significance is the Matlaga paper which described the difference between matched obese patients who either underwent RYGB or did not and the rates of KSD [14•]. 7.65% of those who underwent an operation (355 of 4639) were found to develop KSD compared to 4.63% (215 of 4639) of patients who did not undergo surgery (*p* < 0.0001). Notably, Durrani studied patients undergoing RYGB who were non-stone formers and stone formers prior to the surgery [13]. This study found a greater incidence of KSD in both patients with new KSD (3.2%) and known stone formers (8.8%). Of known stone formers, 32% went on to have recurrence of the disease. This is important because known stone formers who proceed to having a GI procedure could be at significant risk of recurrence.

Two of three studies accounting for obesity as a confounding factor (i.e. controls with matched obese patients who did not undergo surgery) found that post-GI surgery, patients had higher rates of KSD incidence compared to controls (11 to 4.3% and 7.6 to 4.6%) [14•, 18].

In relation to restrictive bariatric procedures, Chen reported a lower KSD incidence rate of 1.2% from 332 banding and 85 sleeve gastrectomies over a follow-up period of 5 years [12]. Conversely, Semins reported a higher KSD incidence rate in obese controls who did not undergo surgery (6%) vs those who underwent gastric banding (1.5%) over a follow-up period of 2.3 years [15].

Overall, there is clear and consistent data indicating that KSD has a greater incidence following bariatric bypass surgery; however, there is less consistent data for gastric banding.

### Inflammatory Bowel Disease and Kidney Stone Disease

There is a lack of studies assessing KSD in IBD patients post-surgery. Overall, it is said that approximately 9–18% of adult IBD patients will develop KSD at some point [6••]. It is thought that the loose stools and malabsorption which accompanies IBD, with or without bowel surgery, are what contributes to the development of symptomatic and asymptomatic KSD in this patient group. For instance, one review stated that IBD patients post-surgery may have a stone prevalence of up to 16%, in comparison to 1.5–5% when they did not undergo any bowel procedure [24]. There is a wide variety of incidence rates of KSD reported in IBD patients in the literature [6••].

It is thought that IBD leads to stone formation by creating a state of hyperoxaluria secondary to malabsorption, lack of oral intake post-surgery, and increased loose stools leading to similar pathogenesis, as discussed above [25].

It is recognised that formation of ileostomy is considered a risk factor for development of KSD. Stones most associated with ileostomy formation consist of uric acid and calcium [26]. In one study, IBD patients with J-pouch were compared to IBD patients with ileostomy and controls. They found that the relative risk of calcium stones was significantly higher in those with ileostomy [6••]. There have been no high-quality studies looking at preservation or resection of large bowel and the effect this has on stone formation in IBD patients. The one study included in this review that compared SB resection only, SB and LB resection, LB resection only, and no procedure found no significant difference in stone incidences (15-32.2%) or time to stone formation [19•]. There was great variability reported, and while the majority of patients were with IBD, the study also included trauma, cancer and obese patients.

### Cancer Related Bowel Surgery and Kidney Stone Disease

The one study solely dedicated to post-operative gastric cancer patients reported that the extent of gastrectomies (rather than the method of reconstruction) and sex for gastric cancer patients serve as independent risk factors for KSD, with total gastrectomies and male sex leading to higher incidence rates of KSD [17•]. This was a good quality study which took place in Japan, where gastric cancer incidence is relatively high, lending itself to a good sample size. The higher incidence of KSD in TGRY compared to DGBI and DGRY might signify increased fat malabsorption with stomach loss. There was no difference between DGBI and DGRY KSD rates, which might mean that the amount of stomach resected is more important than remaining small bowel post-reconstruction.

There is a distinct paucity of papers available regarding KSD and cancer patients who have undergone a GI procedure. This is an important focus of research for the future as the global burden of cancer is on the rise, with GI cancers (stomach, colorectal and liver) among the five most common cancers in both sexes worldwide [27]. Of note is the high mortality associated with GI cancers (accounting for 35.4% of cancer deaths worldwide in 2018) [28]. This may account for the lack of research conducted into patients post-operatively; however, patients selected for therapeutic surgery are presumed to have relatively better outcomes. With surgical resection remaining the primary treatment method for most GI cancers (not including metastatic disease), it would be beneficial to consider the impact of KSD in these patients post-operatively. From a quality of life (QoL) perspective, it is important to prevent KSD through lifestyle and medication modifications where possible, but it could also play a role in choosing which investigation is used for recurrence surveillance [29]. Patients known to be recurrent stone formers might benefit from imaging modalities that can monitor for recurrence and KSD rather than endoscopic modalities alone. Although management of the cancer will always remain a priority for clinicians, awareness of KSD and prevention of it in this patient population will greatly enhance QoL.

### Implications for Clinical Practice

KSD is a costly disease with significant implications for patient morbidity and mortality [30, 31]. Recognising KSD following GI surgical procedures is important for educating medical staff and for optimising clinical management of these patients.

From an educational perspective, it is important to raise awareness among medical staff for diagnostic and consent purposes. It is well-recognised that post-GI surgery patients are often investigated for complications and follow-up by CT imaging. It is important to highlight KSD as a differential diagnosis in post-operative patients presenting with abdominal pain with a convincing clinical history. Examination and bedside tests, such as urine dipstix, may encourage a non-contrast CT scan to help exclude the relatively prevalent KSD in this population. It is important to avoid using contrast where possible as post-operative patients often require numerous scans, whether it be for monitoring or diagnostic purposes (e.g. recurrence of disease), and there is a risk of complications related to the overuse of contrast and CT imaging [32].

Consent is an important aspect of maintaining patient autonomy in surgery. Requirements for legal consent vary from country to country; however, general principles include informing capacitous patients of risks and benefits associated with procedures. A brief snapshot of consent forms found on Google, the search engine, was performed on 27 August 2020 from a UK computer using the search terms “gastric bypass consent”. This informal snapshot identified 14 consent forms from the first 20 search engine results, originating from hospitals in the USA, UK, Canada, Australia and India. Only one consent form included KSD as an explicitly stated risk of the procedure. It should be noted that the other consent forms included “unlisted complications”. It is important to highlight to patients undergoing GI procedures, particularly gastric bypasses where the risk of KSD post-procedure is well-documented, that KSD is a long-term risk of the procedure.

KSD is an important risk factor for renal dysfunction which could have important implications for further management options post-operatively [33]. This is important for IBD and cancer patients where a wide range of medical therapies including chemotherapy are available that patients with chronic kidney disease (CKD) may not be able to access [17•]. The presence of metabolic abnormalities (e.g. hyperoxaluria) may lead to renal dysfunction over time regardless of stone formation as crystal deposition in renal tissue leads to significant injury. It is important to identify and prevent KSD and metabolic abnormalities caused by GI surgery as they are potentially reversible causes of renal dysfunction. An early metabolic assessment for KSD and appropriate medical management might help avoid surgical intervention in these patients.

Management of these post-GI procedure patients should involve investigation and treatment of hyperoxaluria where proven. Options include lifestyle and medication modifications to reduce the amount of total and free enteric oxalate and to reduce urinary excretion of calcium. Oxalate is absorbed from the GI tract, and methods for reducing this include restricting oxalate-rich food (e.g. tea, spinach, bananas) and increased fluid intake. Oral calcium supplements may be considered as an option because they bind with enteric oxalate, reducing the amount of free oxalate being absorbed and excreted renally [33]. There are medications to help reduce urinary calcium excretion and so increase enteric calcium concentrations and reduce enteric free oxalate concentrations. Such medications include citrate preparations and thiazide diuretics [34]. These management options can be combined with traditional KSD lifestyle and medication advice. Fluid intake and electrolyte management in post-surgical patients are particularly important as they are prone to dehydration and electrolyte imbalances due to vomiting/diarrhoea/malabsorption from GI procedures.

Overall, recognising KSD in post-GI surgery patients is important for management of patients. In proven stone formers post-surgery, one might consider referring early on to stone clinics to ensure medications are optimised following blood tests and/or stone analysis.

### Strengths and Limitations of our Study

A key feature of this review is the inclusion of all GI procedures rather than limiting to IBD or bariatric surgery. We found a paucity of evidence for GI surgeries performed for different indications (e.g. cancer, trauma). This would be an important focus of future research. Despite this lack of variety and depth in the literature, the papers that did cover these alternative indications were of moderate to good quality.

The heterogeneity of papers necessitated a narrative summary rather than a primarily quantitative analysis which unfortunately leads to limitations in the certainty of conclusions that may be drawn. The majority of studies conducted retrospective analysis of databases, making studies prone to selection bias. Only 4 out of 12 papers achieved a high level of rigour by matching non-stone formers post-GI surgery to no surgery.

The diagnosis of KSD was done by a variety of methods as outlined previously. In many studies, the timing of performing imaging for patients was not specified, leading to potentially missed cases of patients who developed KSD and passed stones spontaneously. Mukewar was the only study to use both clinical history and formal imaging to ensure those patients were caught [16•]. Of interest, the two Brazilian studies relied on US imaging for diagnosis of KSD [10, 11]. The use of US imaging (rather than CT) as a first line might lead to underdiagnosing KSD in these patient groups, and the highly inconsistent incidence rates support this (1.7% and 17.9%, respectively) [10, 11].

Finally, as with any systematic review, the inconsistent terminology used for procedures and conditions means that articles may have been missed. Every effort was made to include eligible studies, and a great number of databases and resources were reviewed with expansive search terms.

## Conclusion

KSD is an important condition that may be associated with a variety of GI surgeries undertaken for numerous indications. This systematic review serves as an update on the existing literature to provide a more detailed insight as to the incidence rates of KSD developing post-GI surgery. Specifically, we have highlighted the incidence of KSD after a variety of gastric and bowel surgeries for indications including IBD, obesity and cancer.

Where the GI surgery was done for obesity, inflammatory bowel disease or cancer, the risk of KSD ought to be kept in mind during follow-up and prompt urology involvement with metabolic assessment, medical and or surgical management offered as applicable.
